# Intrinsic Disorder as a Biomimetic Design Paradigm

**DOI:** 10.3390/biomimetics11040267

**Published:** 2026-04-12

**Authors:** Thiago Puccinelli, José Rafael Bordin

**Affiliations:** 1 Department of Materials Physics and Mechanics, Institute of Physics, University of São Paulo, São Paulo 05508-090, Brazil; thiagoponogueira@gmail.com; 2 Fachbereich Physik, Universität Konstanz, 78464 Konstanz, Germany; 3 Departamento de Física, Instituto de Física e Matemática, Universidade Federal de Pelotas, Pelotas 96001-970, Brazil

**Keywords:** intrinsic disorder, biomimetic design, intrinsically disordered proteins, biomolecular condensates, liquid–liquid phase separation, soft matter, adaptive materials, molecular self-assembly

## Abstract

Molecular engineering has traditionally followed a structure–function paradigm based on well-defined, folded architectures. However, intrinsically disordered proteins and regions (IDPs/IDRs) reveal that nature also exploits disorder as a functional design strategy. Here, we argue that intrinsic disorder can be understood as a biomimetic design principle for molecular and materials engineering. From a soft matter perspective, IDRs function through statistical ensembles, weak multivalent interactions, and collective behavior rather than fixed structure, with sequence features encoding a molecular grammar that governs phase behavior, viscoelasticity, and responsiveness. These principles closely parallel those found in associative polymers and colloidal systems. Recent advances in coarse-grained modeling, machine learning, and inverse design further enable disorder to be treated as a controllable engineering variable. By reframing intrinsic disorder as a programmable and bioinspired design strategy, this Perspective highlights its potential for the development of adaptive and responsive biomimetic materials.

## 1. Introduction

Biological systems routinely achieve complex and adaptive functionality using molecular components that do not adopt stable three-dimensional structures. Intrinsically disordered proteins and regions (IDPs/IDRs) exemplify this strategy, enabling responsiveness, multivalency, and environmental sensitivity without relying on rigid architectures. Nevertheless, for more than half a century, molecular biology and molecular engineering have been largely dominated by a structural paradigm view of function, in which biological activity is assumed to emerge from well-defined, folded molecular architectures, an idea formalized by Anfinsen and reinforced by decades of experimental and theoretical work on protein folding [1,2]. The resulting sequence–structure–function paradigm has been extraordinarily successful, underpinning major advances in enzymology, molecular recognition, drug discovery, and rational protein design. At the same time, it has implicitly promoted the notion that rigidity and structural specificity are prerequisites for functionality in most molecular design strategies. Importantly, this limitation is not restricted to intrinsically disordered proteins. Even globular proteins rely on conformational fluctuations and exploration of their underlying energy landscapes to perform their biological functions, indicating that a single-structure description is, in general, an approximation rather than a complete representation of molecular behavior.

This picture has been profoundly revised with the recognition of intrinsically disordered proteins (IDPs) and intrinsically disordered regions (IDRs). Once regarded as rare exceptions or experimental artifacts, IDPs are now known to be widespread across proteomes—particularly in eukaryotic organisms—and to play central roles in regulation, signaling, transcription, and cellular organization [3,4,5]. Rather than representing failures of folding, intrinsic disorder is now better understood as an evolutionarily selected strategy, enabling functional plasticity, tunable affinities, multivalent interactions, and rapid responses to environmental and chemical cues. A key and long-recognized feature of many disordered sequences is their high charge density and low hydrophobic content, which places them in the regime of polyampholytes and polyelectrolytes. This physicochemical signature promotes expanded conformations, strong sensitivity to ionic conditions, and sequence-dependent intramolecular interactions, providing a natural link between IDR behavior and polymer physics descriptions based on charge patterning and electrostatic balance. Importantly, these principles are not merely conceptual: recent experimental work has shown that disorder-driven phase separation can be engineered in synthetic systems, enabling the formation of functional biomolecular condensates with tunable properties [6].

Viewed through a physical perspective, this versatility can be traced back to the nature of the underlying free-energy landscapes. Whereas folded proteins typically reside in deep, well-defined minima, IDRs explore shallow and rugged landscapes that support broad ensembles of interconverting conformations [7,8]. These ensembles respond sensitively to changes in concentration, ionic strength, macromolecular crowding, post-translational modifications, and binding partners. Crucially, both experiments and simulations have shown that such fluctuations are not merely random noise, but reproducible and quantifiable physical states. In this way, IDRs behave less like classical molecular machines and, arguably, more like adaptive soft matter naturally connecting protein biophysics to polymer physics and statistical mechanics.

One of the most striking manifestations of this ensemble-based behavior is liquid–liquid phase separation (LLPS) and the formation of biomolecular condensates. LLPS arises when specific intermolecular interactions within a subset of molecules become sufficiently favorable to counterbalance their tendency to remain homogeneously disordered in solution. As a consequence, these molecules preferentially partition into a demixed, condensed phase, leading to their enrichment within the condensate and depletion from the surrounding dilute phase [9,10]. Such interactions reshape the underlying free-energy landscape in a way that allows individual molecules to continuously exchange between coexisting phases while maintaining equal chemical potential across the phase boundary. As a result, no net diffusive flux is established, even in the presence of steep concentration gradients [11]. These biomolecular condensates are formed through weak, multivalent interactions encoded within intrinsically disordered regions, which drive the spontaneous assembly of membraneless compartments with distinct material properties, including viscoelasticity, selective permeability, and dynamic internal organization [12,13,14,15,16,17]. These condensates provide spatial and temporal control over biochemical processes and are increasingly implicated in regulation, stress response, disease [18] and directly promoting oncogenic dysregulation [19,20,21]. Importantly, this behavior is not accidental: sequence-level features encode a molecular grammar that governs collective organization, phase behavior, and material response, establishing intrinsic disorder as a rule-based, rather than structureless, regime of molecular function [14,15,16].

Recent experimental studies have gone a step further by showing that disorder-driven phase behavior can be deliberately engineered. Artificial peptide- and protein-based systems undergo *in situ* phase separation in response to enzymatic activity, osmotic pressure, or chemical signals, forming functional condensates inside living cells and directly modulating biological outcomes [22,23]. Even richer behavior has been observed in systems that spontaneously transition from coacervate droplets to vesicle-like compartments, demonstrating that relatively simple disordered building blocks can give rise to higher-order architectures with emergent material properties [24]. This generality is further supported by synthetic peptide-based systems, where sequence design enables direct control over phase separation behavior and condensate properties, allowing the formation of biomimetic compartments with tunable physicochemical and functional characteristics [25]. The work by Oh et al. has further demonstrated that protein condensates can be engineered with controlled size, stability, and interfacial properties, enabling the design of functional biomaterials with tailored structural and dynamical features [26]. These systems illustrate how biological strategies based on intrinsic disorder can be translated into biomimetic design principles for adaptive and responsive materials. Together, these results highlight disorder-driven self-assembly as a particularly rich and engineerable space for the development of bioinspired compartments and functional materials.

Insights from biomaterials science reinforce this view. Many natural protein-based materials—including elastin, resilin, mussel adhesive proteins, and suckerins—derive their remarkable mechanical, adhesive, and responsive properties from intrinsically disordered or partially ordered architectures organized hierarchically across length scales. Analyses indicate that disorder enables extensibility, toughness, energy dissipation, and environmental responsiveness, offering powerful design principles for bioinspired materials [27].

At the same time, advances in colloidal and nanoscale engineering demonstrate that molecular programmability does not require rigid building blocks. DNA-mediated colloidal assembly, exemplified by spherical nucleic acids and programmable atom equivalents, provides a clear illustration of how flexible, sequence-defined linkers can encode interaction rules that determine symmetry, assembly pathways, and collective behavior [28]. The success of these systems reflects a broader design philosophy in which weak, reversible, and spatially distributed interactions are exploited to generate materials with properties that often surpass those found in nature—a philosophy that closely mirrors the physics of intrinsically disordered regions.

These developments converge with a broader shift in molecular systems engineering toward bioinspired and biomimetic design strategies. Bottom-up synthetic biology, virus-like particle assembly, and bioinspired nanochannels increasingly rely on modular and flexible components capable of self-organization and reconfiguration [29,30]. In parallel, advances in computational modeling and data-driven approaches are redefining molecular design by moving the focus away from single optimal structures toward statistical properties, response functions, and collective behaviors. Recent perspectives highlight the growing role of generative artificial intelligence and inverse design strategies in navigating vast design spaces defined by function rather than form, enabling the systematic exploration of biomimetic systems inspired by the adaptive behavior of disordered biomolecules [31,32,33].

**Figure 1:**
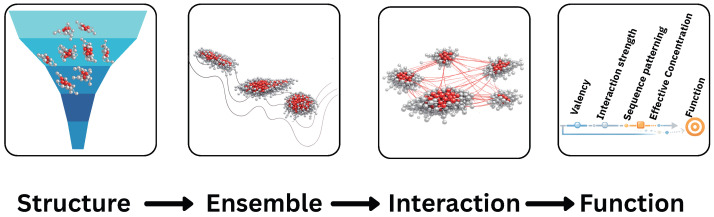
Conceptual illustration of disorder-as-design. From (**left**) to (**right**): a structure-centric paradigm based on unique folds; ensemble-based behavior of intrinsically disordered regions; collective interactions giving rise to emergent organization; and an effective design space in which interaction parameters such as valency, interaction strength, sequence patterning, and effective concentration can be tuned to achieve targeted functional responses.

Taken together, these advances establish intrinsic disorder as a rule-based and bioinspired regime for molecular design, in which collective behavior replaces static structure as the primary carrier of function. Within this framework, IDRs act as tunable substrates whose sequence-encoded interaction rules govern assembly, responsiveness, and emergent function across multiple length scales. Disorder is no longer a limitation to be managed, but a programmable design space that bridges biomolecular physics, soft matter, and materials engineering, offering a versatile foundation for the development of adaptive and responsive biomimetic materials [3,16,34], as schematically summarized in Figure 1.

In this Perspective, we argue that embracing intrinsic disorder as a design principle opens new opportunities for biomimetic materials. By integrating insights from IDP biophysics, biomolecular condensates, bioinspired materials, colloidal engineering, and computational modeling, we outline how intrinsically disordered regions can be deliberately engineered to create adaptive molecular systems, functional condensates, and disorder-inspired colloids and nanomaterials with targeted properties.

## 2. Intrinsic Disorder as Programmable Soft Matter

Unlike folded proteins, IDRs do not impose strong geometric constraints through a rigid tertiary architecture. Instead, they explore broad conformational ensembles whose statistics are shaped by sequence composition and environmental coupling, including electrostatic balance, transient interaction motifs, and regulatory modifications that reshape the underlying free-energy landscape [14,15]. These parameters closely resemble the control variables familiar from polymer physics, where chain flexibility, interaction strength, valency, and sequence heterogeneity determine phase behavior, viscoelasticity, and responsiveness [34,35]. From a polymer physics perspective, IDRs closely resemble associative polymers with transient and reversible interactions [35,36].

This analogy becomes particularly useful in the context of liquid–liquid phase separation (LLPS). Theoretical frameworks originally developed for polymer solutions and networks, including Flory–Huggins theory, random phase approximation approaches, and sticker–spacer models, have been successfully adapted to rationalize condensate formation driven by IDRs [35,37,38,39]. In these descriptions, specific sequence motifs act as effective “stickers”, while more flexible regions function as “spacers” that regulate connectivity, entropy, and material properties [11,40,41]. Importantly, these models provide insight not only into whether phase separation occurs, but also into the dynamical and rheological properties of the resulting condensates. In practical terms, the parameters entering these models can be mapped onto experimentally accessible control variables. Within a Flory–Huggins framework, the effective interaction parameter χ reflects the balance between monomer–monomer and monomer–solvent interactions, and can be tuned experimentally through solvent quality, salt concentration, and sequence composition. In sticker–spacer descriptions, the relevant energy scale associated with sticker–sticker interactions sets the effective attraction strength, while the sticker density along the chain defines the valency and connectivity of the network. Similarly, the overall protein or polymer concentration directly controls the proximity to the phase boundary, as predicted by mean-field and RPA-type approaches. These correspondences provide a direct mapping between model parameters and experimentally tunable design variables in biomimetic systems.

Experimental studies confirm that biomolecular condensates span a wide range of soft matter behaviors, from low-viscosity liquid droplets to viscoelastic and gel-like states [42,43,44,45]. The material properties of these assemblies depend sensitively on sequence features, concentration, and environmental conditions, and they can evolve over time through aging, maturation, or active remodeling processes [13,18]. Such behavior closely parallels that of colloidal gels and polymer networks, where effective interaction potentials determine phase diagrams, mechanical response, and relaxation dynamics [46].

Viewed through an engineering perspective, this polymeric and soft matter description is particularly useful. It implies that IDRs can be designed not by specifying a target structure, but by tuning statistical interaction parameters to achieve desired emergent properties. Relatively modest changes in sticker density, interaction strength, or sequence patterning may shift a system from a homogeneous solution to a viscoelastic condensate, or from a liquid-like droplet to a dynamically arrested network [15]. This kind of tunability mirrors classical strategies in soft condensed matter, where effective interactions are deliberately engineered to control assembly pathways and material properties [46,47].

It is also important to note that IDR-based soft matter often operates far from thermodynamic equilibrium in biological settings. Continuous turnover, enzymatic modification, active processes, and spatial confinement couple molecular-scale disorder to system-level function, allowing condensates to respond adaptively to external stimuli and reorganize internally [13,18]. Recent experiments show that such responsiveness can itself be engineered, for example through enzymatically triggered phase separation or osmotic modulation, reinforcing the idea that disorder offers a route to dynamic control rather than a barrier to predictability [22,23].

The soft matter description of IDRs also creates a direct correspondence to colloidal and nanoscale engineering. In colloidal systems, complex collective behavior is routinely engineered using short-ranged, tunable, and often directional effective interactions [47]. DNA-mediated colloidal assembly is a well-known example, where flexible and sequence-programmable linkers encode interaction rules that determine symmetry, defect tolerance, and reconfigurability [28]. IDRs operate on closely related principles: sequence-defined interaction motifs generate effective potentials that govern assembly, phase behavior, and collective dynamics. This correspondence suggests that inverse design strategies developed in colloidal science may be translated directly to biomolecular disorder.

Computational modeling plays a central role in enabling this translation. Coarse-grained simulations of disordered proteins, informed by atomistic energy landscapes, allow systematic exploration of how sequence features map onto effective interactions and material properties [7,8]. More generally, theoretical frameworks for systematic coarse-graining emphasize that reduced models should reproduce relevant collective observables rather than microscopic detail [48,49]. Within this paradigm, IDRs can be represented as effective polymers or particles interacting via tunable potentials, opening the door to inverse design and optimization strategies for disorder-driven behavior [31,49,50].

At the level of a soft matter perspective, intrinsic disorder is therefore no longer an exception to molecular design, but a regime of material behavior that can be described and manipulated in a systematic way. IDRs follow statistical rules that link sequence features to collective response, phase behavior, and dynamics, in close analogy with associative polymers and colloidal systems. Establishing this physical equivalence is a necessary step before disorder can be more systematically exploited as an engineering substrate.

## 3. Computational and Inverse Engineering Strategies for Disorder-As-Design

Having established ensembles and interaction landscapes as explicit design targets, the remaining challenge is to encode these targets at the sequence level. The central task is no longer the prediction of a single, optimal structure, but rather the ability to control ensembles and their emergent collective behavior. This shift has important consequences for modeling strategies: instead of optimizing geometries, one needs to learn how to shape distributions, correlations, and effective interactions across multiple length and time scales. In practice, this requires a hierarchy of computational approaches that connect sequence features to conformational ensembles, propagate these microscopic rules to mesoscale material properties, and ultimately address the inverse problem of designing sequences or interaction motifs that realize targeted functions [32,33,51,52].

Forward modeling of disordered proteins has reached a level of maturity that allows systematic exploration of sequence–ensemble–function relationships. Residue-resolution coarse-grained (CG) models make it possible to quantify how a small set of low-dimensional sequence descriptors influence not only single-chain properties, but also collective material and dynamical properties [7,8,41,50,53,54,55,56,57,58]. At the same time, careful benchmarking has revealed an important limitation. Agreement at the level of single-molecule observables does not necessarily guarantee accurate prediction of coexistence densities, phase diagrams, or material properties. Different CG parameterizations may reproduce similar chain statistics while encoding distinct physical mechanisms, which suggests that collective observables should be treated as primary design targets rather than as secondary validation metrics [59,60]. This ambiguity highlights the need to prioritize collective observables in model calibration. In particular, phase coexistence properties, such as binodal concentrations, provide direct constraints on the effective interaction parameters governing phase separation. Structural observables in the dense phase, including pair correlation functions and local density fluctuations, further probe the organization of the condensed state. In addition, thermodynamic response functions related to compressibility and concentration fluctuations offer complementary information on interaction strength and correlations. Together, these quantities provide a more stringent and physically grounded basis for parameterizing coarse-grained models than single-chain observables alone.

Consistent with this perspective, minimalist synthetic systems show that key features of intrinsically disordered proteins can be reproduced using simple building blocks [6]. Hybrid constructs combining short peptide “stickers” with flexible polymeric “spacers” undergo liquid–liquid phase separation and form artificial membraneless organelles capable of recruiting biomolecules and enhancing biochemical reactions [61]. These principles extend to cellular contexts, where engineered disordered protein sequences have been used to construct artificial membraneless organelles that improve metabolic efficiency by spatially organizing enzymatic pathways [62]. Peptide-based systems have also been designed to undergo phase separation in response to biochemical triggers, such as enzymatic activity, enabling the formation of functional condensates directly in vivo [63]. More generally, minimal models and biomimetic systems show that controlling a small set of interaction parameters—such as valency, interaction strength, and sequence patterning—is sufficient to reproduce complex assembly pathways and material properties [26,64].

In a physical framework, recent studies based on minimal interaction models have provided a particularly clear demonstration of how collective behavior can be programmed through a small number of microscopic parameters. In this context, association and phase transitions emerge from the competition between short-range attraction, repulsion, and valence constraints, leading to finite-size clustering, internal segregation, and regimes where structural and dynamical properties decouple [65]. Complementary work on patchy particle models shows that modifications to interaction geometry and strength can systematically tune condensate fluidity and dynamics while preserving equilibrium behavior [66], and analyses of generic interaction classes further demonstrate how distinct microscopic forces map onto different phase-separation outcomes and material responses [67]. Consistent with these minimal frameworks, experimental and simulation studies reveal that condensates can form spatially inhomogeneous, network-like fluids whose material properties reflect underlying interaction patterns rather than biochemical identity [68,69,70]. What these results make clear is that controllable collective behavior does not require molecular detail: it emerges once the interaction landscape is properly shaped, placing effective interactions—rather than structure—at the center of rational condensate design. Sure, these minimal models are not intended to replace chemically detailed descriptions, but to isolate the universal interaction mechanisms that govern collective behavior and are otherwise obscured in fully specific systems.

Data-driven and machine-learning approaches provide a complementary route to navigating the very large design space associated with intrinsic disorder [71]. Deep learning predictors of IDR conformational properties enable rapid, proteome-scale screening and hypothesis generation, effectively acting as fast forward models for disorder [72]. In parallel, protein language models trained on large sequence databases are increasingly adapted to annotate disordered regions, identify functional motifs, and infer interaction propensities directly from sequence [73,74,75]. Rather than replacing physics-based simulations, these approaches are better seen as informative priors that can be refined, constrained, and validated by theory and modeling, allowing efficient exploration of sequence space while still retaining physical interpretability [33,51].

A persistent difficulty in disorder-based modeling is the problem of scale bridging. Engineering emergent behavior requires models that remain predictive across changes in concentration, composition, and environmental conditions. Coarse-graining frameworks rooted in statistical mechanics and information theory offer a principled route to constructing reduced models that preserve relevant collective observables, while explicitly quantifying the loss of microscopic detail [49,52,76]. More recent theoretical efforts aim to design CG interactions that remain valid across multiple conformational free-energy surfaces, directly addressing the issue of transferability that is central for predictive design [77,78].

Related works have increasingly made clear that chemical specificity—most notably electrostatics and residue-level interaction heterogeneity—cannot be treated as a secondary detail if one aims to describe phase behavior and material properties of disordered systems across conditions [53,54,59,60,79]. In our view, these studies collectively highlight an important lesson: transferability does not emerge automatically from coarse-graining, but must be built in deliberately, by deciding which collective observables a model is meant to preserve and under which conditions it is expected to remain valid. In a practical modeling workflow, transferability can be incorporated by assigning complementary roles to different levels of description. Machine learning approaches provide a global exploration of sequence space, identifying robust trends and candidate regions across varying conditions. Coarse-grained models are then calibrated to reproduce selected collective observables—such as phase behavior, structural correlations, and concentration-dependent properties—within a defined thermodynamic regime. Finally, targeted atomistic simulations are used to resolve local interaction mechanisms and validate or refine the effective interactions encoded at the coarse-grained level. In this hierarchical strategy, transferability is not assumed but constructed through consistency across scales, with each level constraining the range of validity of the others.

From an engineering point of view, this suggests that disorder-as-design will likely rely on layered modeling strategies rather than on a single universal description [71], combining ML-based screening, CG simulations for collective behavior, and targeted atomistic calculations for chemically specific interactions. Within this multiscale context, the inverse problem naturally comes to the forefront. Instead of asking how a given sequence behaves, one asks which sequence-level features are required to produce a desired ensemble or material response. For intrinsically disordered regions, natural design objectives include target compaction, multivalency patterns, sensitivity to environmental stimuli, phase boundaries, viscoelastic regimes, and selective partitioning. Recent demonstrations of computationally designed IDP variants with prescribed ensemble properties provide a clear proof-of-principle that such objectives can be optimized directly [71]. More broadly, emerging workflows that integrate physics-based modeling, machine learning, and optimization point to a shift from structure-centric to ensemble-centric engineering [32,80].

At a conceptual level, inverse design of disordered sequences closely parallels strategies developed in soft matter and colloidal science. In those contexts, one typically optimizes effective interaction potentials to realize target assemblies or material responses, often using relative-entropy minimization or related objective functions [49,76,81]. Advances in inverse design of pair potentials and soft-material assembly further reinforce the generality of this approach, showing that complex collective behavior can emerge from relatively simple, tunable interaction rules [82,83]. For IDRs, sequence features such as sticker identity, valency, and patterning naturally map onto effective interaction parameters, allowing sequence design to be reframed as a problem of potential design.

A long-standing concern in disorder-based engineering is whether flexibility inherently limits specificity. Recent computational and experimental advances directly challenge this view. Generative and diffusion-based design strategies have shown that binders can be engineered against intrinsically disordered targets by explicitly accounting for heterogeneous conformational ensembles, rather than attempting to suppress them [84,85]. In parallel, sequence-only predictors of chemically specific interactions mediated by disordered regions provide scalable tools for identifying interaction hotspots and functional grammars at the proteome level [86]. In practice, these results indicate that specificity can emerge from shaping an ensemble of compatible microstates, rather than from stabilizing a single conformation [87].

The convergence of transferable coarse-grained models, disorder-aware machine learning, and inverse optimization strategies enables workflows in which ensembles, interactions, and collective responses are treated as explicit design targets. In this framework, intrinsic disorder becomes an operational design variable, supporting the engineering of adaptive biomolecular systems, functional condensates, and disorder-inspired materials within a unified computational approach. While many of the examples discussed here originate from biomolecular systems, the design principles emphasized in this Perspective are not limited to biological implementations. Rather, they define a transferable interaction-based framework that can inform the engineering of synthetic and hybrid systems across molecular and mesoscopic scales.

## 4. Design Rules and Molecular Grammar for Disorder-Inspired Biomimetic Systems

Building on the interaction-centric framework established above, the remaining challenge is to answer the question: if fixed structure is no longer the main object of control, what takes its place as the organizing principle of molecular design? Across protein biophysics, biomolecular condensates, and soft matter, a fairly consistent answer has emerged. Function is not encoded in a unique folded architecture, but rather in a molecular grammar defined by interaction motifs, sequence patterning, and statistical connectivity [3,5,16]. Making this grammar explicit, and learning how to manipulate it in practice, is central to translating disorder from a biological observation into a usable design strategy.

At the level of sequence, a large body of experimental and computational work shows that the collective behavior of intrinsically disordered regions is governed by a relatively small set of tunable features. Charge composition and charge patterning regulate long-range electrostatic interactions and overall chain compaction, while aromatic and hydrophobic residues act as interaction hotspots that control multivalency and network connectivity [14,15]. How these motifs are distributed along the sequence is often just as important as their overall abundance. Spatial patterning determines whether interactions remain mostly transient, give rise to percolated networks, or drive macroscopic phase separation [40,41]. In practice, these parameters rarely act in isolation, but instead combine to define an effective interaction landscape that shapes ensemble behavior. The correspondence between sequence-level features, underlying interaction mechanisms, and emergent material properties is summarized in Table 1.

These disorder-based principles closely resemble long-established strategies in soft matter and colloidal science. In associative polymer networks, for example, the density, strength, and lifetime of stickers determine gelation thresholds, viscoelastic response, and stress relaxation [35,36]. In a similar way, DNA-mediated colloidal assembly relies on flexible and sequence-programmable linkers to encode interaction rules that control symmetry, defect tolerance, and reconfigurability of the assembled material [28,47]. Intrinsically disordered regions play an analogous role at the biomolecular scale, where sequence-defined motifs generate effective potentials that govern assembly, phase behavior, and responsiveness [16].

One clear advantage of disorder-based design is its inherent adaptability. Because IDRs populate broad conformational ensembles rather than a single well-defined structure, relatively small perturbations—such as post-translational modifications, changes in ionic strength, or interactions with binding partners—can produce large functional responses [13,18]. Viewed through an engineering framework, this sensitivity can be exploited to create switchable and stimulus-responsive systems without requiring large structural rearrangements. Engineered condensates that assemble or dissolve in response to enzymatic activity, osmotic stress, or chemical cues provide concrete examples of how disorder can be used to encode conditional behavior [22,23].

It is also important to note that the molecular grammar of disorder does not operate only at the level of individual sequences. Collective organization becomes particularly rich in multicomponent systems, where several disordered regions with distinct interaction rules coexist. Such systems may exhibit selective partitioning, internal structuring, and multiphase behavior, emerging from differences in interaction strengths and affinities rather than from rigid molecular recognition [12,13,16]. In these contexts, specificity is achieved in a statistical sense, through differential partition coefficients and interaction probabilities, offering a robust alternative to classical lock-and-key mechanisms in crowded and fluctuating environments [87].

These observations naturally motivate a modular view of intrinsically disordered regions. Instead of treating IDRs as amorphous segments, they can be decomposed into functional elements—such as stickers, spacers, and regulatory motifs—whose composition and arrangement define system-level behavior [40,41]. This modularity enables rational recombination and reuse, allowing new functional architectures to be assembled by mixing and matching disorder-based motifs. Such an approach fits well with bottom-up synthetic biology and materials-by-design paradigms, where complex functionality emerges from the controlled combination of relatively simple, well-characterized components [29,88].

Instead of focusing on stabilizing a particular structure, disorder-inspired design emphasizes shaping interaction landscapes that generate desired ensembles and collective responses. By prioritizing molecular grammar over geometry, intrinsic disorder provides a flexible and scalable route to engineering adaptive biomolecular systems, functional condensates, and hybrid materials that remain robust across conditions and length scales [31,32].

## 5. Future Directions and Open Challenges

Despite the rapid progress of recent years, several challenges must still be addressed before disorder-as-design can be fully translated into biomimetic materials and molecular engineering strategies. One of the most persistent difficulties is transferability. Design rules and models calibrated under specific concentrations, salt conditions, or component stoichiometries often lose predictive power when extrapolated to new environments, whereas biological systems routinely operate across a wide range of conditions. This limitation highlights the need for coarse-grained and data-driven frameworks that remain robust across heterogeneous environments, as well as for design observables explicitly defined with transferability in mind [52,60]. Addressing this issue will require tighter integration between theory, simulation, and experiment, enabling feedback loops that connect biomolecular insight to the rational design of bioinspired materials.

A second open frontier concerns multicomponent and nonequilibrium behavior, which are hallmarks of living systems and central to biomimetic design. Much of our current understanding of intrinsically disordered regions and biomolecular condensates is grounded in simplified, equilibrium descriptions. In contrast, biological and bioinspired systems often operate far from equilibrium and involve multiple interacting disordered components with distinct interaction rules. Capturing phenomena such as multiphase organization, internal structuring, aging, active remodeling, and spatial patterning remains a major challenge for both theory and simulation [13,16]. Advancing in this direction is essential for translating disorder-based principles into realistic biomimetic platforms, including synthetic condensates, adaptive materials, and artificial cellular systems.

**Figure 2:**
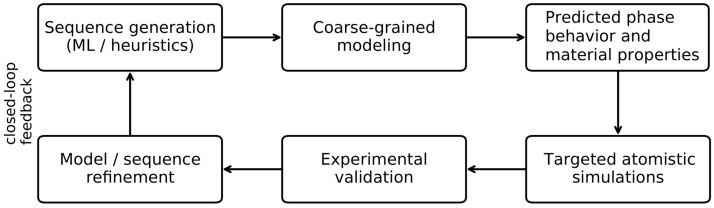
Schematic representation of a closed-loop design workflow for intrinsically disordered systems. Sequence candidates are generated using data-driven or heuristic approaches and evaluated through coarse-grained simulations to predict phase behavior and material properties. Targeted atomistic simulations provide mechanistic insight into local interactions, while experimental validation assesses functional performance. Feedback between these levels enables iterative refinement of both models and sequences.

Finally, there is a clear need for closed-loop design workflows that integrate machine learning, physics-based modeling, and experimental validation within a biomimetic framework. Many of the individual components required for such pipelines already exist, including protein language models, residue-level coarse-grained simulations, inverse design strategies, and generative approaches. What remains largely unexplored is their systematic integration into end-to-end workflows capable of proposing designs, testing them computationally, validating them experimentally, and iterating efficiently [31,32]. Developing such workflows represents a key opportunity for biomimetic molecular engineering, allowing intrinsic disorder to be treated not only as a descriptive feature, but as a controllable and optimizable design variable for adaptive and functional materials. A schematic representation of this closed-loop design workflow is shown in Figure 2.

## 6. Conclusions

Intrinsic disorder reshapes how molecular function and design can be understood. Rather than signaling a breakdown of the structure–function paradigm, intrinsically disordered regions reveal a complementary regime in which functionality emerges from ensembles, weak interactions, and collective behavior. When viewed through the lens of soft matter physics, IDRs can be interpreted as naturally occurring biomimetic systems, whose properties are governed by statistical rules encoded at the sequence level rather than by a single, stable structure.

In this Perspective, we have argued that embracing disorder as a design principle opens new directions for biomimetic molecular engineering. By bringing together insights from protein biophysics, biomolecular condensates, soft matter theory, and computational design, we outlined how intrinsically disordered regions can be deliberately engineered to achieve targeted emergent properties. In this context, ensembles and interaction landscapes are no longer purely descriptive concepts, but actionable design variables that can be harnessed to guide the development of adaptive and responsive systems.

More broadly, disorder-as-design reframes molecular engineering around interaction landscapes and ensemble control, offering a scalable alternative to structure-centric approaches. Instead of focusing on stabilizing static architectures, it emphasizes shaping interaction rules that remain functional across conditions, length scales, and environments. This perspective aligns closely with the goals of biomimetics, providing a conceptual and practical framework for translating biological strategies into the design of adaptive materials, synthetic condensates, and responsive nanoscale systems. In this sense, intrinsic disorder emerges not only as a fundamental feature of biological organization, but also as a powerful and transferable blueprint for next-generation biomimetic materials and molecular technologies.

## Figures and Tables

**Table 1:** Mapping between sequence features and emergent physical properties in intrinsically disordered systems.

Sequence Feature	Physical Effect	Emergent Behavior
Charge composition (net charge)	Electrostatic repulsion/attraction	Chain expansion or compaction; phase boundary shifts
Charge patterning	Long-range electrostatic correlations	Modulation of LLPS propensity; sequence-dependent condensation
Aromatic residues (e.g., Tyr, Phe)	π–π and cation–π interactions	Stabilization of condensates; increased cohesion
Hydrophobic residues	Solvent-mediated attraction	Enhanced phase separation; changes in material viscosity
Sticker density (interaction motifs)	Multivalency and connectivity	Network formation; control of condensate stability
Spacer properties (flexibility/length)	Entropic elasticity	Modulation of chain conformations and phase behavior
Sequence heterogeneity	Interaction diversity	Tunable viscoelastic and dynamic properties

## Data Availability

No new data were created or analyzed in this study. Data sharing is not applicable to this article.
